# Mercurials in the Treatment of Typhoid Fever

**Published:** 1850-12

**Authors:** A. Becquerel


Art. II.—*Mercurials in the treatment of Typhoid Fever.* By M. A. Becquerel. Translated from the *Comptes Rendus,* by David W. Yandell, M.D., of Louisville, Ky.

In 1847 M. Serres presented to the Institute a series of memoirs on the nature and treatment of typhoid fever, in which he directed attention to a new therapeutic method which he believed would prevent the more formidable accidents, and mitigate the more dangerous symptoms of the disease. The treatment consisted in the internal administration of the black sulphuret of mercury (ethiops mineral), in doses varying from twelve to thirty-six grains daily, and in the use of frictions on the abdomen with Neapolitan ointment, in quantities varying from ʒiv. to ℥j. in the twenty-four hours; this treatment being continued eight, ten, or twelve days, or even longer, until the disappearance of all the characteristic symptoms of the disease.

The following are the conclusions stated by M. Serres in the communications referred to.

The employment of the black sulphuret of mercury in the above doses, may almost always be continued eight, ten, or twelve days, without producing salivation, and at the end of this time, when ptyalism is produced, it is in its mildest and most manageable form.

The mercurial frictions over the abdomen always result in the early disappearance of the rose colored spots, and a rapid diminution in the tympanites. Under the combined influence of these two remedies the diarrhea subsides, the pulse falls, the fever grows less, the cephalalgia and delirium are greatly mitigated.

This treatment, it is true, cannot be said to exercise any very great influence on the ordinary duration of typhoid fever, but it unquestionably possesses most valuable powers in preventing unpleasant and fatal complications, and especially in lessening the intensity of the adynamic characters of the disease.

M. Becquerel having temporarily had charge of the ward entrusted to M. Serres, at La Pitié, continued the plan of treatment adopted by the latter gentleman, in typhoid fever, and has furnished the Academy with its results in fifteen cases; and in the paper from which I make this extract, writes in substance as follows:

The whole fifteen cases were very grave and were treated strictly according to the formula of M. Serres, in order that the results—more favorable than those obtained by M. Serres—should be attributed to no other therapeutic agent.

The ethiops mineral was employed from the date of the admission of the patient into the hospital, commencing with eighteen grains, divided into five or six equal doses, and given in pill or powder at regular intervals. If no improvement was manifested in the course of two or three days, the dose of the mineral was increased to twenty-five or thirty grains, and sometimes even thirty-six grains in the twenty-four hours. The medicine was never pushed beyond the last mentioned quantity, and was invariably suspended from the moment convalescence declared itself. The ptyalism was in no case sufficiently severe to oblige the remedy to be prematurely discontinued.

The frictions on the abdomen with the Neapolitan ointment, corresponded in quantity with the doses of the black sulphuret; for instance, with eighteen grains of the mineral, half an ounce of the ointment was used, in two frictions; with twenty-five grains of the sulphuret, three-quarters of an ounce of the ointment were used in three frictions; and, finally, with thirty-six grains of the sulphuret, one ounce of the ointment was applied, also in three frictions.

After each application of the ointment, cataplasms were placed over the abdomen in order to facilitate absorption, and every other day the parts were well washed with soap and water with the same view.

The adjuvants to the mercurials were ice, seltzer water and lemonade for drink; simple, or in cases in which constipation existed, gentle laxative injections; in the ataxic form, of which there were four examples, musk in doses of from five to six grains *per diem*, was combined with the sulphuret. This was discontinued as soon as the delirium and agitation diminished in violence.

Fifteen patients, all attacked with grave typhoid fever, were rigorously submitted to the use of mercurials. There were ten men and five women: the men were aged, 2, sixteen years, 2, seventeen years, 2, eighteen years, 1, twenty years, 2, twenty-two years, 1, thirty-six years. The five women were aged, 1, fifteen years, 1, eighteen years, 1, twenty years, and 2, twenty-one years.

The ten men were attacked with typhoid fever in the following forms: four, with the ordinary abdominal form, attended by stupor, cephalalgia, etc.; five, suffered with the most severe adynamic form; one, with the ataxic form, with delirium and restlessness. The five women presented two examples of the ataxic form, one example of the adynamic, one of the ataxo-adynamic, and one of the ordinary abdominal variety.

The fifteen cases entered the hospital after having been sick for a short time at home. In all, the treatment was commenced the day after their admission.— The following is a description of the effects produced on the principal symptoms and on the general disease:

*The Fever.*—Under the influence of the first dose of the black sulphuret, and of the first frictions, the skin invariably became less hot, less dry, and, in some cases, it was even covered with moisture or perspiration. At the same time the pulse diminished in force and frequency; this effect was produced in every instance, even in the single fatal case which occurred in one of the men, the consequence of an intestinal perforation.

*The Tongue*—at first dry and cracked, and the gums and lips fuliginous, always lost these characters as soon as salivation was produced. Of the fifteen cases, salivation was effected in twelve; twice it did not ensue, and the patients recovered, though in both these cases the tongue remained dry until the entire cessation of the fever. In the case of intestinal perforation, the tongue continued dry throughout the disease. Of the twelve cases in which salivation was produced, it occurred in two on the sixth day of treatment, in three on the seventh day, in four on the eighth day, in one on the twelfth day, and finally, in one on the thirteenth day. With one single exception the salivation was exceedingly slight, lasted four or five days, and required no special treatment. In the instance alluded to, the ptyalism and swelling of the gums were very severe, and lasted for twelve days.

Relative to salivation the following general propositions may be laid down:

1st. In cases of typhoid fever of the ordinary form and of moderate intensity, salivation is produced with greater rapidity, is more severe, and lasts a longer time during convalescence. As a general rule it immediately precedes this latter state.

2d. In cases of greater gravity, salivation requires more time to be produced, is, generally speaking, slight, and does not continue longer than during the first days of convalescence: it precedes but a short time the subsidence of the fever, and constitutes a sign of speedy recovery. In some instances, however, it appears only coetaneously with the cessation of the febrile reaction.

3d. In extremely severe cases, salivation is produced with great difficulty, and so long as it is absent there is much reason to fear more or less unfortunate complications. It is in such cases that we must use for a long time and in large quantities the black sulphuret and mercurial frictions.

4th. In some mild cases salivation does not occur at all. The treatment has invariably been continued in the whole fifteen cases not only till salivation was declared, but until the cessation of the fever, and amelioration of all the symptoms.

The abdominal tympanites, save in the case of intestinal perforation, diminished with astonishing rapidity from the commencement of the treatment—a result which must be attributed to the combined use of the black sulphuret and mercurial frictions.

There were two cases out of the fifteen, in which constipation existed, and was relieved, after the black sulphuret had been tried without effect, by simple injections. In two other cases the black sulphuret produced a slight diarrhea.

In three instances, the diarrhea, characterised at the time of their admission by five or six liquid stools during the twenty-four hours, appreciably diminished as soon as the black sulphuret was administered. Finally, in eight cases, the diarrhea was neither augmented nor diminished, but pursued its course, and diminished only when the other characteristic symptoms of the disease improved.

The mercurial frictions invariably removed, in the course of twenty-four or thirty-six hours after their use, the rose colored spots wherever situated, whether on the abdomen, the immediate seat of the frictions, or base of the thorax, chest, or elsewhere.

Relative to the *cephalalgia*, no sensible effect was noticed in any of the cases; it did not, moreover, constitute in any of them a prominent phenomenon.

In the four cases in which delirium existed, musk was associated with the black sulphuret in doses of from five to six grains, until this symptom disappeared. In the whole four cases, the amelioration was rapid, and five days was the longest term of this complication. In one patient, in which the delirium was so violent as to require the application of the straight-jacket, cold applications were made to the head for three days.

The stupor of the face did not abate until there was an improvement in the other symptoms.

The same may be said of the cough and sibilus. There was not, moreover, it may be remarked, any marked implication of the respiratory organs, in any instance, save that of the patient who died of intestinal perforation on the twentieth day of the disease, and who, at the time of his entry, presented symptoms of a very intense bronchial engorgement. The mercurials did not consequently exercise any direct influence over this symptom.

In no case were there any serious hemorrhages.

The duration of the treatment was, in four cases, seven days, in three cases eight days, in one nine days, in three ten days, in one twelve days, in one fifteen days, in one sixteen days, in one seventeen days; finally, the mean duration of the treatment was ten days.

The minimum of the black sulphuret employed during the treatment was ʒij., grs. vj.; the maximum was ʒvij., grs. viij. The minimum of mercurial ointment employed in frictions was ℥iijss.; the maximum ℥xj., ʒiij.; the mean of the black sulphuret was ʒiij. ℈j. grs. xij. for each, and ℥vijss. of mercurial ointment.

The total duration of the disease varies in a very remarkable degree. One case recovered in twelve days, two cases in thirteen days, three in fourteen days, three in fifteen days, one in sixteen days, one in eighteen days, one in twenty days, one in twenty-one days, and finally, one in twenty-three days. The single fatal case terminated, as has been remarked, on the twentieth day. The mean duration of the disease, that is to say, of the fever, has been, then, sixteen days.

The patient who died of perforation of the intestine was a man of thirty-six years of age, stout and robust, who was admitted into the hospital on the eighth day of the disease; the symptoms of typhoid fever were masked by a very intense bronchitis, which obscured, during the first few days, the principal disease; and was treated for five days—from the 8th to the 12th—by two venesections, ipecacuanha, and a purgative. The bronchial engorgement, though subdued by this energetic treatment, did not admit of the administration of the mercurials before the twelfth day: when given they produced their usual good effects; the different symptoms improved, and the patient was doing well, when, without known cause, on the nineteenth day of the disease, he was seized with symptoms of acute peritonitis, of which he died the following day, the autopsy revealing its cause,—perforation of the intestine.

*Duration of Convalescence.*—Among all the patients it was simple, without complication, and without accident; in one single case, that of a female, who labored under the most intense adynamic form of the disease, an eschar was formed on the sacrum, which required a month to heal. With this exception, and counting from the disappearance of the fever, the patients remained in the hospital from eight to twenty-three days.

**Dec. 1, 1850.**

